# Worldwide Mycotoxins Exposure in Pig and Poultry Feed Formulations

**DOI:** 10.3390/toxins8120350

**Published:** 2016-11-24

**Authors:** Philippe Guerre

**Affiliations:** Sciences Biologiques et Fonctionnelles, Université de Toulouse, ENVT, Toulouse, F-31076, France; p.guerre@envt.fr; Tel.: +33-056-119-3840

**Keywords:** mycotoxin, feed, pig, poultry, exposure, diet, feed substitution

## Abstract

The purpose of this review is to present information about raw materials that can be used in pig and poultry diets and the factors responsible for variations in their mycotoxin contents. The levels of mycotoxins in pig and poultry feeds are calculated based on mycotoxin contamination levels of the raw materials with different diet formulations, to highlight the important role the stage of production and the raw materials used can have on mycotoxins levels in diets. Our analysis focuses on mycotoxins for which maximum tolerated levels or regulatory guidelines exist, and for which sufficient contamination data are available. Raw materials used in feed formulation vary considerably depending on the species of animal, and the stage of production. Mycotoxins are secondary fungal metabolites whose frequency and levels also vary considerably depending on the raw materials used and on the geographic location where they were produced. Although several reviews of existing data and of the literature on worldwide mycotoxin contamination of food and feed are available, the impact of the different raw materials used on feed formulation has not been widely studied.

## 1. Introduction

Mycotoxins are secondary fungal metabolites that can be found in a wide range of raw materials used in human food and animal feed. Although there are hundreds of mycotoxins, regulatory limits or maximum tolerated levels guidelines in food and feed have only been established for a few [1]. Recognition that mycotoxins affect health and productivity in pig and poultry farming has led to legislated maximum tolerated levels (directives) for aflatoxins, and regulatory guidelines (recommended tolerance levels) for ochratoxins and a small number of fusariotoxins. The limits vary with not only type of mycotoxin, animal species, the intended use, raw materials, feed, and diet but also with the regulatory organization or country (Table 1). The European Union (EU) has established guidelines on raw materials and diets with differences linked to age of animal and stage of production (Table 1) [2,3,4]. The Food drug administration (FDA) also established regulatory guidance that varies with the raw material, diet, effect of age and intended use, some of which sometimes differ from EU regulations (Table 2) [5]. Canada generally uses the FDA guidelines although there are some differences for complete diets, and some recommendations for diascetoxycirpenol, T-2 toxin, and HT-2 toxin that are not included in the FDA guidelines [6]. India uses the FDA maximum tolerated levels and regulatory guidelines with some simplifications [7]. 

Although some data about mycotoxins content and contamination in food and feed are available worldwide, it is difficult to identify the exact impact of feed formulation on pig and poultry exposure. The purpose of this review was thus to analyze the factors responsible for variations in the levels of mycotoxins in pig and poultry diets, focusing on the raw materials commonly used to formulate diets with consideration of physiological stage, production, and geographic location of farming. Because not all the data are available on worldwide mycotoxin contamination of raw materials, this review is limited to compounds subject to regulatory or recommended tolerance levels in pig and poultry diets.

## 2. Pig and Poultry Diets

### 2.1. Nutrient Requirements and Feed Formulation

Feed formulation consists of the calculation of the different amounts of raw materials that are necessary to form a diet that supplies all the nutrients required by an animal. Since 1944, the nutrient requirements of swine have been defined by reports of the American National Research Council that provides updated recommendations on phase feeding programs for growing-finishing pigs and sows. The 11th edition published in 2012 also includes computer models to estimate energy, amino acid, calcium, and phosphorus requirements for swine at different life stages [8]. Most of the recommendations used worldwide to define poultry nutrient requirements are those of the American National Academy of Sciences from 1994 [9]. Although the need for an update has been underlined [10], this document provides a large database on components of poultry diets and nutrient requirements. Several tables of composition and nutritional values of produced feed materials found in different countries can be used to complete these recommendations [11].

Nutriment requirements and feed formulation varies with the animal species, age, stage of production, and composition (energy, proteins, mineral, and vitamins) of raw materials available. Because feed costs account for two thirds or more of total live costs in pig and poultry production, particular attention is paid to feed formulation [12,13]. Different strategies are available for feed formulation, most are a combination of energy and protein requirements, which include amino acid digestibility [14,15,16,17]. The presence of indigestible components in raw materials and the use of exogenous enzymes to improve digestibility are also factors that need to be considered in feed formulation [18,19,20,21]. In some cases, feed formulation also include strategies for reducing nitrogen or phosphorus emissions and the environmental impact of the pig and poultry industry [22,23]. Most of the risk of the presence of mycotoxin is “controlled” by the use of feed additives [24,25]. The purpose of this chapter is to present the incorporation rate of raw materials in the pig and poultry diet to estimate the impact feed substitution can have on mycotoxin exposure in these species.

### 2.2. Energy Sources

Table 3 lists the raw materials most widely used as a source of energy in pig and poultry diets. These compounds also provide proteins, mineral, and vitamins but the main reason for their use is energy. The raw materials used as energy sources are the main component of the diets, up to 99% in ducks and geese during force-feeding [26,27]. Cereal grains are the main contributors, but the types of cereal grains used depend largely on their costs and their local availability for animal diet.

Maize is extensively used as an energy source in pig and poultry feed. In America and Europe, the incorporation rate can reaches 50%–80% of the diets. In Asia and Africa, insufficient maize is produced, imports are too expensive, and as a result, maize is not commonly used [28,29].

Sorghum is a widely used source of energy for poultry, especially in hot, drought-prone regions [28]. Older sorghum varieties contained high levels of tannin (>1%), which limits digestibility and use. Low tannin varieties (<0.5%) have 90%–95% of maize energy value. Physical treatment and enzymes can improve sorghum use in poultry diet, up to 50%–60% of incorporation [30,31]. Low-tannin sorghum can be used freely in pig diets, whereas sorghum with high levels of tannin should be used in gradual amounts, starting from 5% in piglets and lactating sows, and reaching up to 20% in the later stages of finishing and for gestating sows [32]. For piglets below 10 kg body weight, it is best to use only low-tannin sorghum and to never exceed 50% of total cereal content.

Pearl millet is a widely used source of energy for poultry in hot, drought-prone regions and in India. Its rate of incorporation is similar to that of maize in pig and poultry diets [31,33,34,35].

Wheat varieties are classified as soft or hard according to their gluten content. Soft wheat varieties are commonly used in Europe and Canada in pig and poultry diets at levels of incorporation of around 30%. Higher levels (80%–85%) have been used in poultry [36,37,38,39,40].

Barley is widely used in animal feed in Europe in pig and poultry diets at levels similar to those of wheat. Pigs fed barley based diets tend to eat more to compensate for the lower energy content of barley compared to maize-based diets [40,41]. Physical treatment or enzyme supplementation can be used to increase the amount of barley that can be incorporated in poultry diet [42].

Oat grains have higher fiber content and lower energy density than barley. Oats can compose up to 90% of the diet of gestating sows, where limiting energy intake is beneficial. To cover energy requirements, oats should not compose more than 20% to 40% of the diet of growing finishing swine and 5% of the diet of small pigs and lactating sows [40,43]. The use of oats in poultry diets is limited [44]. Naked oats have been successfully used as up to 30% of the diet of laying hens, replacing maize, soybean meal, and oil [45]. Enzyme supplementation of oats has been shown to improve growth performance in a variety of poultry species [19,20,46].

Rice is a valuable ingredient of pig and poultry diets. Some rice varieties contains 20% silica which can have adverse effects on poultry performance [47]. Processing rice improves the digestibility of the feed and the productive performance of pigs [48].

Cereal by-products (rice bran, maize gluten feed, wheat feed, confectionary, distillery by-products, and other cereal by-products) are increasingly used in animal feed [49]. These compounds are typically rich in fiber, which is poorly utilized in poultry and is low in energy. Their rate of incorporation can be as high as 10%–15% in some poultry diets and 30% in pig [50].

Fats are a highly concentrated form of energy but inclusion as true fats or oils is limited to 2%–5% of the diet because of their high cost and the risk of rancidity during long-term storage [51].

### 2.3. Protein Sources

The compounds in this section are mainly used for their protein content although they are also a source of energy, vitamins, and minerals (Table 3). They are the second largest component of poultry diets. Amino acid supplementation is usually done to complete the diet. Essential amino acids must be supplied, along with sufficient non-essential amino acids to prevent the conversion of essential amino acids into non-essential amino acids [52].

Soybean meal is the preferred protein source for pig and poultry feed as it contains 40%–50% percent of crude protein, depending on the amount of hulls removed and on the oil extraction procedure used. Although anti-nutritional factors are present in soybean meal, soybean can be used to complement up to 30% of most cereal-based diets [12,53,54].

Rapeseed and canola meals are by-products of oil extraction that can be used as sources of protein in animal nutrition. Rapeseed meal has lower nutritive value than soybean meal because of its lower protein content, higher crude fiber content, and because it contains anti-nutrients such as glucosinolates and erucic acid. The use of rapeseed meal as a substitute for soybean meal is limited in pig and poultry to less than 10% of the diets [55,56,57,58]. Canola was created in the 1970s through plant cross-breeding to remove glucosinolates and erucic acid from rapeseed. Recent literature reviews suggest that performance is affected when more that 50% of soybean meal is replaced by canola meal in pig and poultry diet [59,60,61].

Sunflower meal is also a by-product of the oil extraction process often used in animal feed. It contains 29%–30% protein, 27%–31% crude fiber and 9%–12% lignin [55]. Replacing soybean meal with sunflower meal at high rates is possible in different poultry species [62,63,64,65]. The beneficial effect of enzyme supplementation appears to vary [66,67,68]. Sunflower meal can also be used in pig feed, although some studies suggest that amino acid digestibility was lower than in soybean meal [69,70,71,72].

Cottonseed meal is a rich source of protein (20%–56%) and metabolizable energy that is commonly used for animal feed in Asia and North America [73]. However, its use in pig and poultry diets is limited by the presence of toxic compounds such as gossypol, plus high fiber contents and poor quality protein. No detrimental effect on broiler performance was found when cottonseed meal was used to replace up to 20% soybean meal in the diet [31]. Adequate supplementation with lysine appears to increase performances in broiler chicks fed diets containing extruded cottonseed meal [74]. Similar results have been observed in pigs [70,71,72,75,76].

Animal protein ingredients such dried whey and fish meal can be used to balance the amino acid contents of diets, especially for young animals whose amino acid requirements are high. However, because of their high prices, the rate of incorporation is usually low [12].

### 2.4. Alternative Ingredients

The use of some traditional feedstuffs is based on many years of practice. However, economic rather than nutritive considerations are often the reason for the use of alternative feed ingredients in pig and poultry diets. Fiber and anti-nutritive factors are often present. Cassava roots and other parts of the plant are traditionally used in Africa and Asia as animal feed [77]. Palm kernel meal is used in Southeast Asia, especially in pullet and laying hen diets [28]. Dried sweet potatoes, banana meal, and several other raw materials can be used in some countries but are not listed here because their use is highly specific and very localized [78,79].

## 3. Mycotoxin Contamination

### 3.1. Mycotoxin Analysis

**Figure 1:**
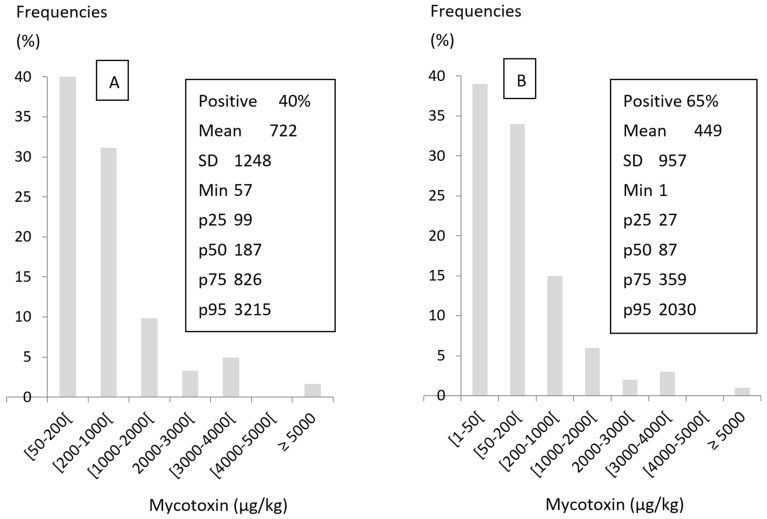
Consequences of a difference in sensitivity for descriptive statistics of mycotoxin contents. (**A**) Method of analysis with a limit of quantitation (LOQ) of 50 µg/kg (old method); (**B**) method of analysis with an LOQ of 1 µg/kg (new method).

Toxicity of mycotoxins and regulations concerning their maximum tolerable level in human food and animal feed have prompted the development of a large number of analytical methods for their identification and quantitation [80,81]. Sampling plays a crucial role in the precision of mycotoxin levels and is usually the main source of variation associated with mycotoxin analysis, causing nearly 90% of the error in some analyses [82,83]. Whatever the method of analysis used, mycotoxin concentration is usually expressed in µg/kg on a dry material basis. Positive results are those observed in samples that are above the limit of detection (LOD) and quantifiable results are those that are above the limit of quantitation (LOQ). Because LOD and LOQ strongly vary with the method of analysis used, care should be taken on comparison of data obtained from different assays [81,82,84,85,86]. The development of more sensitive methods of analysis leads to an increase of the number of positive samples, which considerably modifies the descriptive statistics of the contamination (Figure 1). With highly sensitive methods of analysis, virtually all the large batches that are correctly sampled can be contaminated [83,87]. The observation of a large number of positive samples in a cohort reveals that contamination is frequent, not always that the risk of mycotoxicosis is high. On the contrary, an increase in the number of positive samples generally leads to a decrease in the mean, the median, and the 75th percentile, which does not mean the risk of mycotoxicosis has decreased.

A direct consequence of the marked differences between the methods of analysis is the difficulty in comparing the results of analyses obtained in different surveys. Analysis of the impact of contamination is even more complex when contamination involves several mycotoxins (multi-contamination), which is the most frequent case [88,89]. Because the purpose of this review is to focus on pig and poultry exposure and the factors that influence these variations, only mycotoxins that have a maximum level of contamination in raw materials and feed are taken into account here.

### 3.2. Factors Affecting Mycotoxin Contamination

Mycotoxins are produced by fungi, so virtually all factors that have an impact on fungal development can affect mycotoxin production. Only the factors that need to be taken into account to estimate pig and poultry exposure to mycotoxins are briefly presented here. A simplified approach to fungal development and mycotoxin production is to separate the fungi into two groups: those that invade plants before harvest, commonly called field fungi, and those that invade after harvest, called storage fungi [90].

The factors that are known to have an impact on pre-harvest levels of mycotoxins are the substrate on which the fungus grows and climate conditions. The substrate, i.e., the raw material, is the main factor that influences mycotoxin production by fungi, whose role is discussed in the following section. Climate is the second most important factor known to modify fungal development and leads to variable levels of mycotoxins. For example, in warm humid subtropical and tropical conditions, maize ears are colonized by *Aspergillus flavus* and *A. parasiticus* species, resulting in the formation of aflatoxins. By contrast, in temperate regions, maize is an appropriate substrate for *Fusarium verticillioïdes* and *F. proliferatum* colonization and the production of fumonisins. In temperate regions, ear rot of maize can also be infected by *F. graminearum* that produce deoxynivalenol and other mycotoxins of the trichothecen family. Interestingly, interactions have been reported between *F. verticillioïdes* and *F. graminearum* in maize ears with consequences for mycotoxin accumulation [91]. For these reasons, the geographic origin of a raw material used in a diet will have a major impact on mycotoxins and ultimately, on animal exposure to mycotoxins. In addition to climate, several other factors are also known to modify fungal colonization of plants and the production of mycotoxins. For example, there is a close relationship between insect damage due to the European corn borer and *F. verticillioïdes* infection of maize and fumonisin concentrations. Host resistance and crop management can also affect the level of mycotoxins in maize [92,93].

The substrate, temperature, water availability, and insect damage are known to modify mycotoxin contents during post-harvest storage [90,94]. For example, high levels of aflatoxins in moldy corn infected by *A. flavus* have frequently been found in Africa and Asia as a result of poor handling and storage [95]. A comprehensive review of the biotic and abiotic factors that impact post-harvest fungal ecology and their consequence for mycotoxin accumulation in stored grain is available [94]. The length and conditions of storage should consequently be taken in account when comparing the results of analysis of mycotoxins in the raw materials used to make animal feed; unfortunately this information is rarely available.

Finally, several factors other than annual variations in rainfall and temperature affect mycotoxin levels in plants: not only the geographic origin of the raw material but also the year it was grown will have an impact on mycotoxin levels. Relative importance of the year of analysis, rainfall, and temperatures, on mycotoxins contents is higher for results of contamination that are provided at the scale of a district or a country rather than for results provided at the scale of a continent or part of a continent [96]. Accordingly, the effect of climate change on mycotoxin contamination has been extensively studied [97,98]. However, to date, long-term trends have been difficult to establish as strong yearly variations are observed in worldwide mycotoxin prevalence and contamination levels [88].

### 3.3. Contamination of Raw Materials by Mycotoxins

#### 3.3.1. Maize, Wheat and Soybean Meal

Among the factors that are known to have an impact on contamination by mycotoxins, the substrate, i.e., the raw material on which the fungus grows, is probably the most important. However, because studies on mycotoxin contamination are often done on a large number of food, feed, and commodities and because considerable differences are found regarding the type and prevalence of mycotoxin contamination in the different regions of the world, the contribution of each raw material to these contaminations is not easy to establish [88,89,96,99,100,101,102,103]. Also, several contamination studies give the percentage of positive samples with the maximum concentration observed, but descriptive statistics on the results observed that are necessary for the estimation of animal exposure as a function of the different raw materials used are not always given. Table 4 lists the number of samples with the percentage of positive, the median value, the third quartile value and the maximum value observed in a worldwide survey conducted on three different raw materials (maize, wheat and soybean meal) that are among the most frequently used in pig and poultry diets [100]. A recent overview that includes more analyses revealed no major differences in worldwide distribution of the mycotoxins analyzed and those listed in Table 4 [89]. However, because international exchanges are so common, special attention should be paid to possible differences between the geographic location of sampling and geographic origin of the raw material. For example, most of the wheat used in the EU is produced locally, whereas imports of maize account for 15%–20% of total maize used and imports of soybean account for more than 70% of soybean used [29]. Discussion of the levels reported is based on the most constraining regulatory guideline previously reported (Table 1 and Table 2).

Concerning aflatoxins in cereals, the frequency of positive samples was highest in Southern Europe, South Asia, and South-East Asia (average values of positive samples >30%), in agreement with what is known about the distribution of this toxin across the world [89]. However, analysis of the frequency of positive results is not enough to analyze contamination, and descriptive statistics are necessary [103]. Maize contamination above 20 µg/kg but below 100 µg/kg was observed in South-East Asia and South Asia at the 50th percentile. At the 75th percentile, contamination of maize that exceeded 20 µg/kg but was still below 100 µg/kg was also observed in North America and Northern Asia. At this percentile, contamination of maize from South-East Asia and South Asia largely exceeded 100 µg/kg. Very high aflatoxins levels, above 1000 µg/kg were only observed among maximum values in Asia. By contrast, contamination of wheat and soybean by aflatoxins was much lower. Levels above 20 µg/kg, but never above 100 µg/kg, were only observed among the maximum values in some area of the world. In southern Europe, although 43% of wheat samples tested positive, the maximum value was only 6 µg/kg.

Worldwide contamination of maize, wheat and soybean meal by ochratoxin A at levels higher than 250 µg/kg were reported at the 75th percentile in maize in South America and at the maximum concentration in maize and in South Asia and in wheat in Central Europe.

Deoxynivalenol contamination varied considerably depending on the raw material analyzed and where is was produced. The maximum recommended levels in raw materials also varied remarkably (Table 1 and Table 2). DON levels above 5000 µg/kg were observed at the maximum in different raw materials in North America, Central Europe, North Asia, South-East Asia, and Oceania, but only once at the 75th percentile in wheat in Oceania. Because of the attention paid to deoxynivalenol in the pig, it was interesting to compare its levels at lower concentrations than the recommended concentrations in maize and wheat. In North America and Northern Asia, maize and wheat showed similar levels of contamination at the 75th percentile. In South America, South-East Asia, and Oceania contamination of wheat was 5–10 fold higher than that of maize, both at the 75th percentile and at the maximum concentration. Maize was more contaminated than wheat in central Europe whereas wheat was more contaminated than maize in Southern Europe. Finally, except for wheat in Oceania, worldwide contamination of maize and wheat at the 75th percentile was less than 2000 µg/kg. Soybean meal contamination by DON was 2–10 fold lower than that of maize and wheat.

Maize was frequently contaminated by fumonisins, with more than 50% of samples tested positive whatever the geographic origin of the maize, except North America. However, fumonisin levels above 20,000 µg/kg were only observed in North America, South America, and Northern Asia and none exceeded 60,000 µg/kg. At the 75th percentile, the level of fumonisins was never above 5000 µg/kg whatever the origin of the maize. Contamination of wheat and soybean meal by fumonisins is rare, the number of positive samples was always below 20% except in Central and Southern Europe. Contamination at the 75th percentile was usually lower than 1000 µg/kg except in wheat in South America.

Maximum concentrations of zearalenone in maize never exceeded 3000 µg/kg except for the maximum found in North America and Northern Asia. Zearalenone in wheat was always below 2000 µg/kg except for the maxima found in South-East Asia and Oceania. Comparison of the zearalenone levels in cereals revealed that higher levels in maize than in wheat were only observed in Northern Asia and Oceania whereas levels were similar in the other parts of the world. Contamination of soybean meal by zearalenone was 2–10 fold lower than that of maize and wheat. 

Complementary data from Northern Europe revealed that contamination of animal feed by aflatoxins and fumonisins was lower than that observed in Central and Southern Europe, whereas contamination by ochratoxin A, deoxynivalenol, and zearalenone was within the same range [96]. High levels of mycotoxins are known to be present in food in Africa, although the raw materials used for animal feed are not frequently analyzed, and most studies focus on human exposure to aflatoxins. Not only aflatoxins but also fumonisins have been frequently reported in some African countries, with high levels in maize. Maximum concentrations of aflatoxins and fumonisins were 355 µg/kg and 20,000 µg/kg in Ghana and Zambia, respectively [104]. Levels of ochratoxin A in cereals in Africa varied considerably depending on the location of the sample, respective maximum concentrations of 112 and 2106 µg/kg being observed in cereals in Tunisia-Morocco and Ethiopia. By contrast, contamination by deoxynivalenol and zearalenone appears to be low in both human food and animal feed in Africa [104].

Taken together, contamination data suggested that except for aflatoxins, mycotoxins levels above the strictest regulatory limits or guidelines were only observed at the 75th percentile for ochratoxin A in maize in South America and for deoxynivalenol in wheat in Oceania. By contrast, contamination of maize by aflatoxins at higher levels than the EU regulatory level was observed at the 50th percentile in South-East Asia, South Asia and probably Africa and at the 75th percentile in North America and Northern Asia.

#### 3.3.2. Other Raw Materials

It is more difficult to find data on worldwide contamination of other raw materials than maize, wheat, and soybean meal. Sorghum appears to be a possible substrate for the toxinogenic fungi that are found in maize [105]. Aflatoxins, ochratoxin A, and zearalenone were found in sorghum, and contamination by aflatoxins increases with the length of storage [106]. Pre-harvest contamination by aflatoxins was also reported, but although mycotoxins are present in preharvest sorghum grains, the problem may not be as severe as in preharvest corn [107]. Sorghum contamination by T2-toxin appears to be high [108].

Only a few data are available on millet contamination. This raw material can be contaminated by *Aspergillus* strains that produce aflatoxins but also toxinogenic *Penicillium* and *Fusarium* strains [109,110]. Low concentrations of aflatoxins, deoxynivalenol, and zearalenone have been found in some studies, suggesting that millet may be less sensitive to fungal infection and mycotoxin production than maize [111].

Barley contamination by fungi and mycotoxins appears to be similar to that in wheat, but conflicting results are reported, suggesting high variation in barley breed sensitivity to fungal infection and mycotoxin accumulation [112,113,114,115,116,117]. Like for other raw materials, long term storage in inappropriate conditions was shown to increase mycotoxin contents in barley [115,118,119].

Contamination of oats by mycotoxins also appears to vary. In some studies, oats were less contaminated (both in frequency and concentration) by deoxynivalenol, zearalenone, and T2 toxin than maize, wheat and barley [120]. By contrast, other studies reported high levels of fusariotoxins in oats, sometimes higher that those observed in wheat [121]. As for other cereals, environmental conditions have notable consequences for the accumulation of T2 toxin and HT2 toxin in field oat grains [122]. However, it is not clear whether the variations in the contamination reported were due to differences in sensitivity of the breed of oats to fungal infection, as found to be the case in barley.

The profile of mycotoxins in rice appears to be similar to that in wheat, but usually at lower concentrations [108,115,123]. Like for other raw materials, a recent review revealed marked variations in contamination of rice worldwide [124]. However, maximum levels of aflatoxin contamination were always below 20 µg/kg except in Turkey, India, and Nigeria where respective maximum levels of 21, 308, and 1642 µg/kg were measured. Ochratoxin A was always below 25 µg/kg except in Morocco, Turkey, and Nigeria, where respective maximum levels of 47, 81; and 341 µg/kg were measured. Only a small number of results on deoxynivalenol in rice are available, and all were below 1000 µg/kg. Trace levels of fumonisins were found in some samples. Zearalenone was always notably lower than 2000 µg/kg (the maximum level of 1169 µg/kg was found in Nigeria).

Mycotoxin contamination of cereal by-products varies considerably. The fate of mycotoxins during cereal milling and ethanol or beer production was recently reviewed [89], so this point will not be detailed here. Together, the physical-chemical properties of the mycotoxin, its location in the different part of the grain, and the process used affect partitioning. As a consequence, respecting the recommended maximum tolerable levels of mycotoxins in cereals (Table 1 and Table 2) does not guarantee these levels will be respected in cereal by-products.

Contamination of oils by mycotoxin is generally recognized to be low. Although high levels of aflatoxins have been reported in crude rice bran oil, it has been demonstrated that the refining process considerably reduces contamination (maximum level of 956 µg/L and 28 µg/L in crude and refined oil, respectively) [125]. Another study on vegetable oil revealed that although aflatoxin contamination was found in 28% of samples, the maximum level observed was <1 µg/kg [126]. Similar results were observed in sunflower, sesame and groundnut oils [127,128]. These results agree with a calculation model on the fate of contaminants during the refining of vegetable oils, which suggested that aflatoxins are decomposed during refining [129]. By contrast, peanut butter prepared traditionally in Sudan for human consumption was highly contaminated by aflatoxins, (highest value of 170 µg/kg) confirming the important role of the refining process in reducing aflatoxin contamination [130]. Zearalenone was shown to reach relatively high levels in refined maize oil (more than 100 µg/kg), but only a small number of samples were analyzed [131].

Only a few data are available on mycotoxin contamination in grapeseed, canola, and sunflower meals because these meals are by-products of the food oil industry and most of the analyses are conducted on the oils themselves, whereas specific methods of analysis are needed to avoid interactions with the matrix in the results. The presence of aflatoxin B1 in sunflower oils and mustard suggests that this mycotoxin could be present in sunflower and grapeseed meals [132,133]. In the same way, because zearalenone was found in rapeseed and maize oils it could also be present in the meals, but probably at a lower concentration than that observed in maize [131]. Alternariol, alternariol monomethyl ether, and tenuazonic acid were found in rapeseed and sunflower meals, but the consequences of these levels for pig and poultry health are difficult to establish [134,135]. Studies on mycoflora and mycotoxin production in oilseed cakes during on-farm storage suggested that toxinogenic strains of *Aspergillus fumigatus* can develop and produce gliotoxin [136]. Old studies on mustard and mustard products also suggested that toxinogenic strains of *Fusarium oxysporum* can grow on Brassicaceae and produce diaxetoxyscirpenol, T-2 toxin and zearalenone [137].

Cottonseeds and cottonseed by-products are naturally contaminated by *Aspergillus*, which produces aflatoxins. A study conducted from 1997 to 2001 in Texas (USA) revealed marked yearly variations in cottonseed contamination. The highest level of contamination was measured in 1999, with aflatoxins averaging 112.3 µg/kg and an average of 65.7% of truckloads of cottonseed with aflatoxin contents equal to or above 20 µg/kg [138]. Date of harvest, the length of time before ginning, and the storage conditions have a marked impact on the level of aflatoxin in cottonseed [139,140,141]. In Egypt, levels of up to 200 µg aflatoxins/kg of cottonseed cakes have been reported, whereas no ochratoxin A or zearalenone were detected [142]. Similar results, but with lower levels of aflatoxin, were found in the UK, with six samples out of 21 contaminated by aflatoxins at a rate of between 20 and 99 µg/kg and no samples contaminated by ochratoxin A and zearalenone [143]. In Columbia, although a large number of samples tested positive for aflatoxins, the maximum level measured was 11.4 µg/kg [144]. Because of the carcinogenic properties of aflatoxins, several studies were conducted with the aim of reducing aflatoxin contamination of cottonseeds. A study of the effect of the extrusion temperature showed that aflatoxin levels were reduced by an additional 33% when the cottonseed was extruded at 160 °C compared to at 104 °C [145]. Transgenic Bt cotton was developed with expected reduced susceptibility to aflatoxin contamination. Unfortunately the Bt cultivars were not resistant to an increase in aflatoxins after boll opening and large quantities of aflatoxins can form during this period [146]. New strategies to develop resistance to aflatoxins in cottonseed and maize have emerged. They include the use of strains of *A. flavus* in fields that are not toxinogenic and the development of cultivars resistant to aflatoxin through overexpression of resistance genes [93,147].

The traditional use of alternative ingredients in pig and poultry diets is based on many years of practice, but because these alternative ingredients are only used locally it is very difficult to find data on the level of mycotoxins they may contain. Most of the countries in which these raw materials are used are developing countries, where aflatoxins are the main mycotoxins that should be taken into account [104].

Although a carry-over of some mycotoxins into meat and fat has been demonstrated, no regulatory limits apply to these commodities destined for human consumption, the only regulations are for aflatoxin M1 in milk and milk by-products [2,5]. Most metabolic and toxicokinetic studies conducted with fusariotoxins suggest that these mycotoxins are weakly absorbed and that the resulting metabolites are less toxic than the parent compound [148]. Consequently, protein such as dried whey, fish meal, and fat of animal origin that are incorporated in pig and poultry feed can be considered safe with respect to their mycotoxin content.

## 4. Pig and Poultry Exposure

Since animal exposure to mycotoxins mainly occurs through feed, and contamination of the raw materials used for feed formulation varies considerably, the risk of exceeding regulatory guidelines will vary with the mycotoxin, the formulation, and the geographic origin of the raw materials used. Different methods of evaluation of risk and different scenario of exposure can be used [149]. All cannot be compared here, but it seems of interest to highlight the influence that the geographic origin of the raw material can have on mycotoxin contamination of feed at different stages of production and the impact the most common feed substitutions can have on these levels.

### 4.1. Impact of Age and Production

Nutrient requirements vary with the age and the animal concerned and these variations have consequences for feed formulation. Table 5 lists example of reference diets that could be used for pig production [150]. Contamination data in Table 4 made it possible to calculate theoretical mycotoxin levels in feed using three different contamination scenarios: median, the 75th percentile, and maximum contamination. Calculations were made for five geographic areas that are representative of the worldwide contamination reported in Table 6. Mycotoxin exposure linked to dried whey, synthetic amino acids, and vitamins was assessed as null. Of course, the probability of feed made of different raw materials all with the maximum level of contamination is very low and this scenario can thus be considered as the worst case scenario. By contrast, special care should be taken when feed exceeds the recommended guidelines when raw materials contaminated at the median level are used. Because EU guidelines are stricter than FDA guidelines, the calculated levels of mycotoxins in feed were compared to the EU guidelines.

An aflatoxin level in feed >20 µg/kg was observed for the diet calculated with raw materials at the median of contamination in South-East Asia and nearly all the diets calculated with raw materials at the 75th percentile of contamination in North America and in Northern Asia. By contrast, levels generally below 10 µg/kg where found in South America and Southern Europe. Although all the diets with the worst cases of contamination of raw materials exceeded 20 µg/kg, marked variations in levels were observed depending on the geographic location at which the raw materials were produced. Aflatoxin levels above 1000 µg/kg were observed in Northern Asia and South-East Asia, whereas the levels were around 500 and 200 µg/kg in North and South America, respectively, and below 50 µg/kg in Southern Europe. Interestingly the levels of aflatoxins calculated in the diet also varied with the stage of production. In the diet for pigs weighing 10–30 kg, aflatoxins were around two fold lower than in feed for 80–160 kg pigs and sows, which was due to using high quality and purified ingredients.

Ochratoxin A above 50 µg/kg feed was observed in feed destined for pigs weighing 80–160 kg and gestating sows calculated with raw materials at the 50th percentile of contamination in South America and all the diets that used raw materials at the 75th percentile. By contrast, ochratoxin A above 50 µg/kg but below 100 µg/kg was only observed in worst case scenarios in South-East Asia and never in North America, Southern Europe, and Northern Asia. The level of ochratoxin A was around two fold lower in the diet used for 10–30 kg pigs when high levels of ochratoxin A were present in the raw materials, whereas only slight differences were found between diets at the lowest level of contamination.

Calculated contamination of the reference diets by fusariotoxins varied considerably depending on the mycotoxin studied. Deoxinivalenol was never observed at levels above 900 µg/kg in South America and was only observed in worst case scenarios in North America, Southern Europe, and South-East Asia. The use of raw materials at the 75th percentile of contamination in Northern Asia also led to diets that contained more than 1000 µg/kg of deoxynivalenol in 80–160 kg pigs and sows, but not in piglets. Fumonisins at levels above 5000 µg/kg were only observed in worst case scenarios on contamination of the raw materials, whereas in most cases, the use of raw materials at the 75th percentile of contamination led to diets that contained less than 3,000µg fumonisins/kg. Zearalenone near and above 100 µg/kg in feed destined for 10–30 kg pigs was always observed in diets that used raw materials at the 75th percentile of contamination. Zearalenone above 250 µg/kg in 80–160 kg pigs and in sows was also observed in Northern Asia in diets that used raw materials at the 75th percentile of contamination. In all other parts of the world, the use of raw materials at the 75th percentile of contamination led to diets that contained less than 250 µg zeearalenone/kg feed. Zearalenone above 500 and sometimes at 5000 µg/kg was found when the raw materials used were contaminated at the maximum level. 

The calculation of the levels of mycotoxins in the different diets used for pig production can be compared with data on contamination directly measured in animal feed [88,96,99,100,101]. Generally, data measured in feed agree with calculated levels, but the calculation also revealed differences linked to the stage of production that do not appear in the results of feed analysis. Feed used for piglets appeared to be two fold less contaminated than feed used for 80–160 kg pigs and sows, especially when high mycotoxin levels were present in the raw materials used. This can be explained by the relatively low level of maize used in the diet of 10–30 kg pigs, maize being the main contributor to mycotoxin exposure in the reference diets used for the calculation. In the same way, maize can represent 99% of the diet in duck and geese during force feeding. Because of the high amount of diet provided, this production is highly exposed to fumonisins and risk of toxicity [151]. 

Consequently, replacing maize in feed could have a strong impact on the level of mycotoxins in the final diet. The consequences of feed substitution are analyzed in more detail for broiler diets.

### 4.2. Impact of Feed Substitution

Although maize and soybean meal are the reference materials for pig and poultry feed, it is interesting to see the impact of alternative formulation on the mycotoxin contents of the diet. Table 7 shows an example of feed substitution that can be used in broilers whose performances have been compared in the literature [152]. Calculation of mycotoxin contents in these diets using three different levels of contamination of the raw materials used are reported in Table 8 for the five representative geographic areas. Mycotoxin exposure linked to other sources of energy than maize or wheat that were added to the feed used was not taken into account because of the low contamination of tallow and sunflower oil and their low rate of incorporation into the feed. Exposure to mycotoxins linked to mineral, amino acids, and vitamins was considered to be zero. 

Aflatoxins in the diets of broilers was higher than 20 µg/kg in feed that use maize + soybean meal contaminated at the 50th percentile in South-East Asia and in North America and North Asia for diets made of raw materials at the 75th of contamination. By contrast, diets above 20 µg/kg were only observed in the case of the most contaminated maize and soybean in South America and Southern Europe. Interestingly, aflatoxins never exceeded 20 µg/kg feed in diets that used wheat + soybean meal. Ochratoxin A in the diet was only higher than 100 µg/kg feed in South America in maize based feed that used raw materials at the 75th percentile of contamination.

Calculated levels of fusariotoxins in feed never exceeded the maxima recommended by the EU in poultry, except for a few worst case scenarios in some areas of the world. However, the consequences of replacing maize with wheat varied considerably depending on the toxin concerned. Calculated levels of fumonisins strongly decreased whereas a threefold increase in deoxynivalenol levels in broiler feed was found in South America, Southern Europe, and South-East Asia and this substitution has no consequences in North America and Northern Asia. Only slight consequences of replacing maize with wheat were found for the level of zearalenone in feed.

Finally, replacing maize with wheat was always advantageous in terms of mycotoxin in broiler diets. The same results are likely to occur in pigs. The impact of other feed substitutions can be extrapolated from the risk of mycotoxin contamination in the different raw materials listed in Table 2. Replacing maize by sorghum or millet will probably result in a decrease in the level of mycotoxins in feed. The impact of using barley and oats instead of maize is more difficult to analyze because of the wide variation in some mycotoxins found in these raw materials. However, it is likely that the final level of mycotoxins in feed will be near the level observed when maize is replaced by wheat, but complementary data on levels of T2 toxins and HT2 toxins in cereals are necessary. The impact of replacing maize by rice should be analyzed case by case because of the few available contamination data and the high level of contamination by aflatoxins sometimes observed. The same can be said for the use of cereal by-products, as mycotoxin contamination varies considerably depending on the physical-chemical properties of the mycotoxins and the process used [89].

The consequences for the levels of mycotoxins in feed of replacing soybean with raw materials that are source of proteins will also vary depending on the raw materials used. Because contamination of grapeseed, canola, and sunflower meal by mycotoxins has been shown to be low, their use in feed as a substitute for soybean meal will not have a major effect on total mycotoxin levels in feed. Also, as previously found in piglets, the use of protein of animal origin reduces the level of mycotoxins in feed. By contrast, because cottonseed meal was found to be highly contaminated by aflatoxins, at least in some areas of the world, care should be taken when considering it as a possible replacement for soybean meal, especially when maize was not the principal source of energy in the feed in countries where highly contaminated cottonseed meal was produced.

In conclusion, even feed substitution is not easy to conduct, economic considerations often lead to the use of different raw materials, including non-conventional ingredients, in pig and poultry diets. Although the toxicity of mycotoxins in pig and poultry has been known for several years and regulatory guidelines exist for several of these compounds, no worldwide data enable comparison of the mycotoxins levels in the different feeds used at the different stages of production. Calculation of theoretical levels using data of contamination of the main raw materials used in feed revealed differences depending on the raw materials used, their geographical origin and their intended use. The risk of going beyond the limits laid down in mycotoxin guidelines in pig and poultry feed is higher for aflatoxins in maize based diets, and this risk will increase if soybean meal is replaced by cottonseed meal, at least in some parts of the world. By contrast, the use of wheat reduced the calculated level of aflatoxins in pig and poultry diets. Calculated levels of zearalenone above the maximum recommended were observed in maize based pig diets, but a beneficial effect of replacing maize by wheat is unexpected for this mycotoxin. By contrast, the risk to have diets containing levels of zearalenone above the maximum recommended is weak due to the high tolerable level of this toxin in poultry.

Taken together, this suggests that the choice of the raw materials used during feed formulation has major consequences for exposure to mycotoxins, and should be analyzed with care, especially in pig diets. Complementary data on mycotoxins levels in raw materials that are not commonly used in feed is needed to avoid any risk of overexposure when unusual feed substitutions are used. 

## Figures and Tables

**Table 1:** European Union regulatory levels ^a^ and established guidelines ^b^ on mycotoxins in raw materials and in pig and poultry diets [2,3,4].

Mycotoxin	Feed Materials	Maximum Levels, µg/kg
^a^ Aflatoxin B1	All feed materials	20
	Complete feedstuffs for pigs and poultry (except young animals)	20
	Other complete feedstuffs	10
	Complementary feedstuffs for pigs and poultry (except young animals)	20
	Other complementary feedstuffs	5
^b^ Deoxynivalenol	Feed materials	-
	Cereals and cereal products with the exception of maize by-products	8000
	Maize by-products	12,000
	Complementary and complete feedstuffs with the exception of:	5000
	Complementary and complete feedstuffs for pigs	900
^b^ Zearalenone	Feed materials	-
	Cereals and cereal products with the exception of maize by-products	2000
	Maize byproducts	3000
	Complementary and complete feedstuffs	-
	Complementary and complete feedstuffs for piglets and gilts (young sows)	100
	Complementary and complete feedstuffs for sows and fattening pigs	250
^b^ Ochratoxin A	Feed materials	-
	Cereals and cereal products	250
	Complementary and complete feedstuffs	-
	Complementary and complete feedstuffs for pigs	50
	Complementary and complete feedstuffs for poultry	100
^b^ Fumonisin B1 + B2	Feed materials	-
	Maize and maize products	60,000
	Complementary and complete feedstuffs for:	-
	Pigs	5000
	Poultry	20,000
^b^ Sum T-2 and HT-2 toxin	Cereal products for feed and compound feed	-
	Oat milling products (husks)	2000
	Other cereal products	500
	Compound feed	250

**Table 2:** Food drug administration mycotoxin regulatory guidance in pig and poultry diets [5].

Mycotoxin	Intended Use/Class of Animal	Grain, Grain by-Product, Feed or Other Products	Maximum Levels, µg/kg
Aflatoxin B1	Immature animals	Maize, peanut products, and other animal feeds and ingredients, excluding cottonseed meal	20
	Animals not listed above, or unknown use	Maize, peanut products, cottonseed, and other animal feeds and ingredients	20
	Breeding swine and mature poultry	Maize and peanut products	100
	Finishing swine 100 pounds or greater in weight	Maize and peanut products	200
	Swine or poultry, regardless of age or breeding status	Cottonseed meal	300
Vomitoxin *	Swine	Grain and grain by-products not to exceed 20% of diet	5000
		Complete diet	1000
	Chickens	Grain and grain by-products not to exceed 20% of diet	10,000
		Complete diet	5000
Fumonisin **	Swine	Maize and maize by-products not to exceed 50% of the diet	20,000
		Complete diet	10,000
	Poultry being raised for slaughter and hens laying eggs for human consumption	Maize and maize by-products not to exceed 50% of the diet	100,000
		Complete diet	50,000
	All other species or classes of livestock	Maize and maize by-products not to exceed 50% of the diet	10,000
		Complete diet	10,000

* 88 percent dry matter basis, ** Dry weight basis.

**Table 3:** Raw materials used in pig and poultry feed and risk of mycotoxin contamination.

Raw Material	% in Diet	Mycotoxins ^n^
Maize	0–60 ^a^	High levels of Afla, OTA, DON, FB, ZEA contamination possible. Marked geographic variations.
Sorghum	0–60 ^b^	Few data available. Same mycotoxins as maize but at lower rates.
Millet	0–60	Few data available. Could be relatively resistant to fungal infection compared with other cereals.
Wheat	0–30 ^c^	Contamination by OTA, DON, FB, ZEA possible, with marked geographic variations. Usually weakly contaminated by Afla.
Barley	0–30 ^d^	Same mycotoxins as wheat, with marked variations depending on the breed.
Oats	0–90 ^e^	Same mycotoxins as wheat, marked variations. T2 and HT2 can be high.
Rice	0–30	Same mycotoxins as wheat with lower concentrations of mycotoxins except Afla and OTA, which can be high.
Cereal by-products	0–30 ^f^	Physicochemical properties of mycotoxins, location in the grain, and process used influence partitioning.
Oil or fat	<5	Usually low in refined oils.
Soybean meal	0–40 ^g^	Low level contamination by Afla, OTA, DON, FB, ZEA. Marked geographic variations.
Rapeseed and canola meals	0–20 ^h^	Indirect evidence for the presence of Afla, ZEA, DON, alternariol, tenuazonic acid and gliotoxin at levels near that of soybean meal. Complementary data required.
Sunflower meal	0–40 ^i^	
Cottonseed meal	0–40 ^j^	High level of Afla observed in some parts of the world.
Animal protein	<5 ^k^	Very low contamination.
Alternative ingredients	0–80 ^l^	Sparse data, Afla the most frequently reported. Complementary data required.
Other ingredients	<1–15 ^m^	Not contaminated

^a^ Can increase to 99% in ducks and geese due to force feeding; ^b^ Replacing more than 50% of maize can reduce performances; ^c^ Can be increased to 80%–90% in some diets; ^d^ Fiber content limits use for poultry; ^e^ High fiber content limits use for poultry. Energy requirements influence the rate of incorporation in pig diets; ^f^ Usually less than 15% in poultry, 30% in pig; ^g^ Rate of incorporation varies with oil extraction; ^h^ High fiber content and anti-nutrients limit incorporation of rapeseed to 10%. Higher proportions of canola can be used; ^i^ High fiber content but no anti-nutrients; ^j^ High fiber content and toxic compounds (gossypol); ^k^ Mainly dried whey and fish meal; ^l^ Great number of raw materials, rate of incorporation varies with cost of production and local availability; ^m^ Amino acids, vitamins, mineral, feed additives; ^n^ Afla: aflatoxins; OTA: ochratoxin A; DON: deoxynivalenol; FB: fumonisins; ZEA: zearalenone; High level = found at higher levels than the guidelines in Table 1 and Table 2 at the 75th percentile, low level = never found at higher levels than the guidelines in Table 1 and Table 2.

**Table 4:** Worldwide occurrence of mycotoxin contamination ^a^ in maize, wheat and soybean meal (adapted from Rodrigues et Naehrer, 2012) [100].

**Mycotoxin**	**Raw Material**	**North America**	**South America**	**Central Europe**	**Southern Europe**
Afla ^b^	Maize	10.1–**62**–**920** (375; 26%)	1.8–4.9–**273** (809; 25%)	1.5–1.8–3 (16; 31%)	4.0–12–**44** (42; 36%)
	Wheat	4.1–6.6–9.0 (15; 20%)	2.6–2.6–3.0 (40; 3%)	1.6–2–2 (13; 31%)	1.6–1.8–6 (14; 43%)
	Soybean meal	2.0–2.0–2.0 (74; 1%)	1.0–1.0–1.0A (60; 8%)	1.2–1.2–1.3 (8; 38%)	1.8–2.7–**21** (23; 22%)
OTA ^c^	Maize	2.3–3.1–18 (126; 10%)	71–**275**–**355** (147; 12%)	2.4–2.5–3 (21; 10%)	9.3–29–46 (31; 29%)
	Wheat	0.8–0.8–0.8 (2; 50%)	33–39–43 (11; 45%)	3.8–5.4–331 (22; 23%)	0.7–0.7–1.0 (13; 8%)
	Soybean meal	4.6–5.2–6 (18; 17%)	1.0–10–10 (51; 10%)	21–21–21 (3; 33%)	1.2–1.3–1.3 (22; 18%)
DON ^d^	Maize	565–931–**24,900** (390; 79%)	172–241–939 (322; 17%)	716–1576–**26,121** (535; 72%)	523–705–3851 (59; 47%)
	Wheat	600–976–**7000** (25; 76%)	906–1006–2520 (17; 53%)	514–960–**49,000** (436; 55%)	716–1864–3505 (24; 38%)
	Soybean meal	187–734–**5500** (45; 18%)	197–292–428 (55; 29%)	450–494–741 (43; 21%)	339–428–908 (25; 24%)
FB ^e^	Maize	490–1161–**22,900** (466; 39%)	2008–3890–**53,700** (807; 92%)	684–4504–7680 (30; 60%)	1407–3266–11,050 (48; 90%)
	Wheat	(7; 0%)	1407–1561–1715 (40; 5%)	246–348–450 (9; 33%)	151–538–925 (10; 30%)
	Soybean meal	(46; 0%)	274–295–315 (60; 5%)	(2; 0%)	95–511–5088 (21; 29%)
ZEA ^f^	Maize	86–168–**4787** (395; 29%)	87–222–1800 (321; 43%)	79–155–849 (379; 39%)	166–276–1546 (52; 21%)
	Wheat	275–394–513 (16; 13%)	73–232–393 (32; 47%)	65–123–336 (256; 12%)	(17; 0%)
	Soybean meal	51–142–144 (50; 10%)	81–130–807 (53; 34%)	36–46–56 (31; 6%)	(23; 0%)
**Mycotoxin**	**Raw Material**	**North Asia**	**South-East Asia**	**South Asia**	**Oceania**
Afla ^a^	Maize	7.0–**36**–**4687** (447; 32%)	**97.0**–**206**–**2601** (330; 71%)	**96.0**–**312**–**2230** (108; 82%)	3.0–4.0–5.0 (11; 18%)
	Wheat	3.3–3.6–20 (76; 7%)	1.0–1.0–1.0 (40; 3%)	ND	2.0–5.0–**30** (109; 5%)
	Soybean meal	2.8–2.9–3 (36; 6%)	1.0–3.3–74 (109; 22%)	2.0–3.5–7 (16; 63%)	1.0–1.0–1.0 (3; 67%)
OTA	Maize	1.4–4.1–19 (420; 10%)	3.0–6.3–80 (218; 12%)	7.4–15–**400** (107; 27%)	1.2–1.2–1.2 (11; 9%)
	Wheat	1.0–2.0–7 (67; 22%)	3.9–5.7–30 (40; 30%)	ND	1.6–3.7–4 (108; 8%)
	Soybean meal	1.6–4.4–19 (33; 24%)	2.4–5.6–21 (105; 16%)	14–23–46 (16; 56%)	3.1–3.1–3.1 (3; 33%)
DON	Maize	640–1444–**15,073** (477; 92%)	182–352–4805 (218; 45%)	190–349–1150 (106; 22%)	179–214–249 (11; 27%)
	Wheat	426–1279–**5331** (75; 87%)	199–1483–**41,439** (40; 65%)	ND	719–**5870**–**49,307** (109; 48%)
	Soybean meal	107–202–314 (37; 38%)	228–285–973 (105; 18%)	249–251–259 (16; 31%)	150–150–150 (3; 33%)
FB	Maize	1519–3594–**23,499** (443; 75%)	1033–1720–19,289 (326; 83%)	541–796–6196 (108; 74%)	2344–4023–5438 (11; 64%)
	Wheat	298–471–874 (73; 11%)	172–232–292 (40; 5%)	ND	196–216–1196 (109; 12%)
	Soybean meal	316–319–321 (35; 6%)	228–285–973 (109; 4%)	(16; 0%)	(3; 0%)
ZEA	Maize	176–435–**7446** (470; 67%)	97–206–2601 (319; 20%)	79–174–1099 (108; 9%)	626–939–1251 (11; 27%)
	Wheat	48–82–465 (72; 42%)	53–217–**6641** (40; 40%)	ND	180–351–**23,278** (115; 28%)
	Soybean meal	31–42–398 (34; 35%)	38–53–70 (105; 15%)	(16; 0%)	150–150–150 (3; 33 %)

^a^ Descriptive statistics are: Median—75th percentile—Maximum (number of samples analyzed; percent of positive); Detailed information on the sampling protocol and methods of analysis used can be found in the original manuscript of Rodrigues et Naehrer [100]; ^b^ Afla: aflatoxins; data in bold: above 20 µg/kg (maximum regulatory level, EU, see Table 1 for details); ^c^ OTA: ochratoxin A; data in bold: above 250 µg/kg (maximum recommended level, EU, see Table 1 for details); ^d^ DON: deoxynivalenol; data in bold: above 2000 µg/kg (maximum recommended level, FDA, see Table 2 for details); ^e^ FB: fumonisins; data in bold: above 20,000 µg/kg (maximum recommended level, FDA, see Table 2 for details); ^f^ ZEA: zearalenone; data in bold: above 3000 µg/kg corn or 2000 µg/kg wheat (maximum recommended level, EU, see Table 1 for details).

**Table 5:** Example of reference diets that could be used for pig production (adapted from Holden et al., 1996) [150].

Ingredient (g/kg)	Growing Pig (kg)		Sow	
	10–30	80–160	Gestation	Lactation
Maize	358	766	863	688
Soybean meal	460	210	100	275
Dried whey	150	0	0	0
Other ingredients ^a^	32	24	37	37

^a^ Minerals, amino acids, vitamins.

**Table 6:** Calculated mycotoxin levels in feed using the median, the 75th percentile, and the maximum contamination listed in Table 5 for the four reference diets listed in Table 6. Data in bold exceed EU guidelines for mycotoxins in feed (Table 1).

World area	Production		Afla ^b^	OTA ^c^	DON ^d^	FB ^e^	ZEA ^f^
North America	Pig ^a^	10–30	4.5–23.1–330	2.9–3.5–9.2	288–671–11,444	175–416–8198	54.2–126–1780
		80–160	8.1–47.9–705	2.7–3.5–15	472–867–20,228	375–889–17,541	76.6–159–3697
	Sow	Gestation	8.9–53.7–794	2.4–3.2–16.1	506–877–22,039	423–1002–19,763	79.3–159–4146
		Lactation	7.5–43.2–634	2.8–3.6–14	440–842–18,644	337–799–15,755	73.2–155–3333
South America	Pig ^a^	10–30	1.1–2.2–98.2	25.9–90.9–341	152–221–533	845–1528–19,370	68.4–139–1016
		80–160	1.6–4–209	54.6–187–721	173–246–809	1596–3042–41,200	83.7–197–1548
	Sow	Gestation	1.7–4.3–236	61.4–209–811	168–237–853	1760–3387–46,375	83.2–205–1634
		Lactation	1.5–3.6–188	49.1–169–649	172–246–764	1457–2757–37,032	82.1–189–1460
Southern Europe	Pig ^a^	10–30	2.3–5.5–25.4	3.9–11–17.1	343–449–1796	547–1404–6296	59.4–98.8–554
		80–160	3.4–9.8–38.1	7.4–22.5–35.5	472–630–3140	1098–2609–9533	127–211–1184
	Sow	Gestation	3.6–10.6–40.1	8.1–25.2–39.8	485–651–3414	994–2388–10,045	143–238–1334
		Lactation	3.2–9–36	6.7–20.3–32	453–603–2899	994–2388–9002	114–190–1064
Northern Asia	Pig ^a^	10–30	3.8–14.2–1679	1.2–3.5–15.5	278–610–5541	689–1433–8560	77.3–175–2849
		80–160	6–28.2–3591	1.4–4.1–18.5	513–1149–11,612	1230–2820–18,068	141–342–5787
	Sow	Gestation	6.3–31.4–4045	1.4–4–18.3	563–1266–13,039	1343–3134–20,312	155–380–6466
		Lactation	5.6–25.6–3226	1.4–4–18.3	470–1049–10,457	1132–2560–16,256	130–311–5232
South-East Asia	Pig ^a^	10–30	35.2–75.3–965	2.2–4.8–38.3	170–257–2168	475–747–7353	52.2–98.1–963
		80–160	74.5–159–2008	2.8–6–65.7	187–330–3885	839–1377–14,980	82.3–169–2007
	Sow	Gestation	83.8–178–2252	2.8–6–71.1	180–332–4244	914–1513–16,744	87.5–183–2252
		Lactation	67–178–1810	2.7–5.9–60.8	188–321–3573	773–1262–13,538	77.2–156–1809

^a^ Body weight (kg); ^b^ Afla: aflatoxins; data in bold: above 20 µg/kg feed (maximum regulatory level, EU, see Table 1 for details); ^c^ OTA: ochratoxin A; data in bold: above 50 µg/kg feed (maximum recommended level, EU, see Table 1 for details); ^d^ DON: deoxynivalenol; data in bold: above 900 µg/kg feed (maximum recommended level, EU, see Table 1 for details); ^e^ FB: fumonisins; data in bold: above 5000 µg/kg feed (maximum recommended level, EU, see Table 1 for details); ^f^ ZEA: zearalenone; data in bold: above 100 or 250 µg/kg feed in piglets and adults, respectively (maximum recommended level, EU, see Table 1 for details).

**Table 7:** Example of replacing maize by wheat that can be used in broilers (adapted from Marquardt et al., 1994) [152].

Ingredient (g/kg)	Maize/Soybean	Wheat/Soybean
Maize	628.3	0
Wheat	0	676.5
Soybean meal	300	200
Other energy source ^a^	20	70
Other protein source ^b^	10	10
Other ingredients ^c^	41.7	43.5

^a^ Tallow, sunflower oil; ^b^ Soybean concentrate; ^c^ Minerals, amino acids, vitamins.

**Table 8:** Calculated mycotoxin levels in feed using the median, the 75th percentile, and the maximum of contamination listed in Table 5 for the four reference diets listed in Table 8. Data in bold exceed EU guidelines for mycotoxins in feed (Table 1).

World Area	Feed ^a^	Afla ^b^	OTA	DON	FB	ZEA
North America	C + S	6.9–39.6–579	2.8–3.5–13.1	411–805–17,295	308–729–14,388	69–148–3051
	W + S	3.2–4.9–6.5	1.5–1.6–1.7	443–807–5836	0–0–0	196–288–376
South America	C + S	1.4–3.4–172	44.9–154–593	167–239–718	1344–2533–33,834	79–178–1373
	W + S	2–2–2.2	22.5–28.4–31.1	652–739–1790	1007–1115–1223	66–183–427
Southern Europe	C + S	3.1–8.3–33.9	6.2–18.6–29.3	430–571–2692	913–2205–8469	104–173–971
	W + S	1.4–1.8–8.3	0.7–0.7–0.9	552–1347–2553	121–466–1643	0–0–0
Northern Asia	C + S	5.2–23.5–2946	1.4–3.9–17.6	434–968–9565	1049–2354–14,861	120–286–4798
	W + S	2.8–3–14.1	1–2.2–8.5	310–906–3669	265–382–655	39–64–394
South-East Asia	C + S	61.2–130–1656	2.6–5.6–56.6	183–307–3311	717–1166–12,411	72–145–1655
	W + S	0.9–1.3–15.5	3.1–5–24–5	180–1060–28,228	162–214–392	43–157–4507

^a^ C + S: maize + soybeal meal; W + S: Wheat + soybean meal; ^b^ Afla: aflatoxins; data in bold: above 20 µg/kg feed (maximum regulatory level, EU, see Table 1 for details); ^c^ OTA: ochratoxin A; data in bold: above 100 µg/kg feed (maximum recommended level, EU, see Table 1 for details); ^d^ DON: deoxynivalenol; data in bold: above 5000 µg/kg feed (maximum recommended level, EU, see Table 1 for details); ^e^ FB: fumonisins; data in bold: above 20,000 µg/kg feed (maximum recommended level, EU, see Table 1 for details); ^f^ ZEA: zearalenone; data in bold: above 3000 or 2000 µg/kg in corn and wheat, respectively (maximum recommended level, EU, see Table 1 for details).
